# Between Violence and Addiction: The Complex Journeys of Resilient Women

**DOI:** 10.1192/j.eurpsy.2025.935

**Published:** 2025-08-26

**Authors:** S. Matmati, E. Khelifa, C. Bey, A. Mtiraoui

**Affiliations:** 1 razi hospital, manouba; 2 farhat hached hospital, sousse, Tunisia

## Abstract

**Introduction:**

Violence against women is a significant public health issue with devastating consequences for victims. It’s a complex and often hidden phenomenon rooted in deep-seated gender inequalities. Faced with distress and trauma, some victims may develop self-medication behaviors, such as substance use.

**Objectives:**

This descriptive study aims to characterize the experiences of violence among 53 women who sought help from the ATL, MST SIDA, and ATIOST associations.

**Methods:**

Data was collected through individual interviews with each of the 53 participants. An interview guide was developed to cover different types of violence (physical, psychological, sexual, economic), their frequency, intensity, and their consequences on substance use, using the DUDIT questionnaire.

**Results:**

The sample consisted of women with an average age of 36.04 ± 10.31 years, ranging from 18 to 58. The results show that all participants experienced multiple and recurrent forms of violence, primarily psychological (insults, denigration, control, isolation) and physical (hits, slaps, threats). Sexual violence, although less frequently reported, was also identified in 32%. The most common type of violence was physical (77%), mainly occurring within the couple (49%). 72% of women presented a substance use disorder. The most commonly used substances were cannabis (53%), followed by benzodiazepines (41%), and pregabalin in third position (36%).

**Conclusions:**

This descriptive study highlights the complexity of the experiences of violence faced by women seeking help from harm reduction associations. The results underscore the urgent need to address this issue and develop tailored responses to the specific needs of these women. Future research could explore in greater detail the risk and protective factors associated with violence against women, as well as the long-term effects of this violence on mental and physical health.

**Disclosure of Interest:**

None Declared

